# Controversies in Ocular Myasthenia Gravis

**DOI:** 10.3389/fneur.2020.605902

**Published:** 2020-11-30

**Authors:** Amelia Evoli, Raffaele Iorio

**Affiliations:** ^1^Fondazione Policlinico Universitario A. Gemelli IRCCS, Rome, Italy; ^2^Department of Neuroscience, Università Cattolica del Sacro Cuore, Rome, Italy

**Keywords:** neuromuscular junction, acetylcholine receptor antibodies, muscle-specific kinase antibodies, autoimmune disease, ophthalmoparesis

## Abstract

Myasthenia gravis (MG) with symptoms limited to eye muscles [ocular MG (OMG)] is a rare disease. OMG incidence varies according to ethnicity and age of onset. In recent years, both an increase in incidence rate, particularly in the elderly, and a lower risk for secondary generalization may have contributed to the growing disease prevalence in Western countries. OMG should be considered in patients with painless ptosis and extrinsic ophthalmoparesis. Though asymmetric muscle involvement and symptom fluctuations are typical, in some cases, OMG can mimic isolated cranial nerve paresis, internuclear ophthalmoplegia, and conjugate gaze palsy. Diagnostic confirmation can be challenging in patients negative for anti-acetylcholine receptor and anti-muscle-specific tyrosine kinase antibodies on standard radioimmunoassay. Early treatment is aimed at relieving symptoms and at preventing disease progression to generalized MG. Despite the absence of high-level evidence, there is general agreement on the efficacy of steroids at low to moderate dosage; immunosuppressants are considered when steroid high maintenance doses are required. The role of thymectomy in non-thymoma patients is controversial. Prolonged exposure to immunosuppressive therapy has a negative impact on the health-related quality of life in a proportion of these patients. OMG is currently excluded from most of the treatments recently developed in generalized MG.

## Introduction

The impairment of neuromuscular transmission (NMT) in myasthenia gravis (MG) is due to loss of acetylcholine receptors (AChRs) and end-plate alterations caused by autoantibodies (Abs). The AChR, the muscle-specific tyrosine kinase (MuSK), and low-density lipoprotein receptor-related protein 4 (LRP4) are the main Ab targets. Extracellular proteins, like neuronal Agrin and collagen Q (ColQ), have recently been recognized as additional antigens (1, 2). In clinical practice, patient subgrouping based on disease-specific Abs is a prerequisite for personalized management (3).

Anti-AChR Abs induce MG through complement activation, AChR cross-linking, and internalization, and, to a lesser extent, by interfering with ACh binding (1). MG with AChR Abs affects around 85% of patients. It has a bimodal incidence pattern with a peak in young women and a larger peak in elderly men and is associated with thymus hyperplasia and thymoma, both playing a role in autoimmunization against AChR (3). On clinical grounds, AChR-MG shows broad variability in weakness severity and extension.

Anti-MuSK Abs are found in 30–40% of AChR-negative patients, with high prevalence in women.

Clinical phenotype is dominated by cranial and bulbar weakness (4). MuSK Abs are mostly IgG4 that interfere with the protein function inhibiting MuSK activation and leading to reduced AChR clustering (1). Anti-LRP4 Abs are detected in a proportion of AChR and MuSK-negative [double seronegative (dSN)] patients, generally in association with mild disease (5, 6), and can be also found in some AChR and in some MuSK-positive cases (5). Anti-LRP4 Abs are IgG1/2 with a potential to activate complement (7). Abs to Agrin (6, 8) and to ColQ (9) have been found so far in few AChR/MuSK/LRP4-negative MG patients, and the associated clinical aspects are not defined. Lastly, some patients, often with juvenile onset and limited disease, do not have detectable serum Abs.

Dysfunction of ocular motility is common in MG and very few patients fail to experience ptosis or diplopia at some point of their disease. The term “ocular MG” (OMG) refers to the disease clinically confined to extrinsic ocular muscles (EOMs). Hereinafter, we review OMG pathophysiology and clinical aspects and discuss issues that are still controversial in its management.

## Pathophysiological and Clinical Aspects

Gaze control requires the precise and sustained activity of the oculomotor system and, in normal individuals, EOM contraction is stable under high rate motoneuron firing (10). EOMs have a unique biological organization with different compartments and six distinct fiber types according to innervation (singly and multiply innervated), metabolic arrangement, and protein expression pattern (10, 11). EOM increased susceptibility to MG can be related to structural and molecular properties different from those in other striated muscles. In EOMs, neuromuscular junctions (NMJs) often show underdeveloped postsynaptic folding (12), with a decreased content of AChRs (13). In addition, low expression of complement regulators, as the decay-accelerating factor (DAF) (14), can increase NMJ vulnerability to the effect of complement-activating AChR Abs and, possibly, LRP4 Abs. Lastly, it is well-known that EOMs express both the adult (α2β*εδ*) and the embryonic (α2β*γδ*) isoforms of the AChR (11). While the relevance of the fetal AChR as Ab target is unclear, its functional characteristics may foster susceptibility to MG. Fetal AChR has a longer open time and higher affinity for agonists (15) and, in a recent study, was found to recover more slowly from desensitization than the adult isoform (16). Such characteristics may reduce EOM adaptability to high-rate innervation and lead to impairment of NMT (16).

OMG should be suspected in patients with painless ophthalmoparesis and intact pupillary reflexes. Symptom fatigability, fluctuations in severity, and a remitting–relapsing course increase the likelihood of OMG diagnosis. Initial manifestations may consist of unilateral ptosis or diplopia due to weakness of a single EOM (17–19). Nonetheless, at the first examination, most patients have ptosis and diplopia with multiple muscle pareses (17–20). Weakness of the orbicularis oculi (which is a facial muscle), although uncommon at presentation (20), is frequent in the later course of the disease (18). AChR Ab-positive and dSN patients share a similar clinical pattern (19). Ptosis is usually asymmetrical, with rapid fluctuations and shifting from one eye to the other. EOMs can be involved in different combinations with a broad variability of unconjugated pareses. Complete external ophthalmoplegia occurs rarely and mostly in chronic disease (18). In patients with MuSK, Abs ocular symptoms tend to be less evident, often consisting in symmetrical gaze limitations with transitory diplopia and bilateral, largely symmetrical, ptosis (21–23). The pattern of ocular dysfunctions associated with anti-LRP4 other Abs has not been described.

## Incidence and Prevalence

MG epidemiology has changed over the last decades with a steady increase in incidence (24–26) and prevalence rates (27, 28), particularly among elderly males. From recent data, it seems that these changes also include OMG.

It is generally accepted that, among adult Caucasians, more than 50% of MG patients present with ocular symptoms. The majority of these cases eventually develop generalized MG (GMG), most often within 2 years from onset, and up to 20% remain affected by OMG (29, 30). In a recent population-based survey, the annual incidence of OMG was 1.13/100,000 (31) at twice the rate previously reported (32), and contemporary studies have consistently shown an increased proportion of males with late-onset disease among incident cases (19, 31, 33). OMG prevalence depends on the generalization rate, which is related to several factors, such as disease duration, treatment, and, above all, ethnicity and age at onset. In Asian countries, particularly in China, a high proportion of patients present in childhood and remain affected with OMG (34, 35), and, irrespective of ethnicity, progression to GMG is more rare in children with prepubertal onset than in adults (35–37). Presence of thymoma (38), signs of NMT failure in limb muscles on electrophysiological testing (39, 40), detection of AChR (31, 41) and MuSK Abs (42), and increased serum levels of microRNA miR-30e-5p (43) were found to be associated with increased risk of secondary generalization. A protective role of immunosuppression was found in some studies (41, 44, 45), but was not confirmed by others (46).

Overall, generalization rate appears to be lower in current studies than in earlier reports based on immunosuppression-naïve patients. Moreover, in subjects treated early with steroids, disease progression may be delayed and become evident after treatment tapering or withdrawal. Recent data support this possibility showing that conversion time can be considerably longer than previously reported (47).

## Diagnosis

OMG is easily suspected in patients with fluctuating asymmetric ptosis and diplopia caused by involvement of multiple EOMs, as very few conditions can mimic such a pattern. On the other hand, the diagnosis can be tricky when ocular symptoms can be due to single nerve paresis or fatigability is not obvious. OMG confirmation relies on serological, electrophysiological, and bed-side tests.

### Serological Testing

AChR and MuSK Ab detection by radioimmunoassay (RIA) is highly specific, as AChR Abs are rarely found in subjects with other diseases or thymoma without MG (1, 48), and MuSK Abs have never been reported in non-MG patients. When MG is clinically suspected, AChR Abs are tested first, and MuSK Abs are assayed in AChR-negative cases. The detection of either Ab confirms the diagnosis, with no actual necessity for electrophysiological or clinical tests.

The sensitivity of AChR Abs in OMG is generally thought to be around 50% (19, 30). However, recent studies reported positivity rates higher than 70%, particularly in male patients (31, 49). These results strengthen the value of AChR Ab standard assay and warrant confirmation. Conversely, there have been very few reports of MuSK-positive OMG (22, 23), although MuSK Ab prevalence in this population has not been systematically investigated.

More recently, the development of sensitive cell-based assays (CBAs), in which Abs bind to antigens concentrated on cell membranes, has expanded the serological diagnosis of MG. Disease-specific “clustered AChR” Abs were reported in 16–45.8% of dSN patients (50–52) and, in a study (52), were strongly associated with prepubertal onset and OMG. In addition, MuSK Abs were detected by an IgG-specific CBA in 8% of dSN sera and, interestingly, 38% of these patients had OMG (53). Although these results are encouraging, it must be considered that CBAs require specific skills and facilities and are not largely available.

With different assays, LRP4 Abs were detected at variable rates (from 18.7 to 2.9%) in dSN patients and, in these studies, OMG frequency ranged 22–53% (5, 54). LRP4 Ab testing has the same limitations and, apparently, lower specificity than AChR and MuSK detection by CBA (55).

Methodological standardization and studies involving large cohorts are needed to establish the diagnostic yield of new Abs (56). At present, these assays should be reserved for dSN patients with positive results on electrophysiological testing or responsive to cholinesterase inhibitors (ChE-Is).

Abs to cortactin have been described in 9/38 dSN-MG patients with mild generalized or purely ocular disease (57). Cortactin, an intracellular muscle protein expressed at the NMJ, contributes to the stabilizations of AChR clusters (58). Abs to cortactin are likely not pathogenic and are not diagnostic of MG. Their possible role as marker of OMG (57) needs confirmation.

### Electrophysiological Studies

In MG, low-rate repetitive nerve stimulation (RNS) typically elicits a >10% decrement of the compound muscle action potential between the first and the fourth or fifth stimuli, and single fiber-EMG (SF-EMG) shows an abnormally increased jitter and, when NMT impairment is severe, intermittent blocking of the second potential. Electrophysiology diagnostic yield depends on testing weak muscles, although SF-EMG can detect an increased jitter in subclinical MG (59).

RNS has limited sensitivity in patients with OMG. In different studies, positivity rates ranged from 16.7 to 44% (19, 60–62), consistently associated with high specificity (63). SF-EMG, when performed in the orbicularis oculi muscle, was found to be 79–100% sensitive for the detection of OMG (19, 63–66), but it is time consuming and not largely available. An altered SF-EMG is commonly found in other disorders of NMT like Lambert-Eaton myasthenic syndrome (LEMS) and botulism, and both low-rate RNS and SF-EMG are frequently positive in congenital myasthenic syndromes (CMS). However, these diseases rarely manifest with purely ocular symptoms. On the other hand, finding an increased jitter in conditions that can closely mimic OMG, as chronic progressive ophthalmoplegia, incomplete Miller-Fisher syndrome (MFS), and ptosis following botulinum toxin injection, may complicate the diagnosis (67).

Repetitive ocular vestibular evoked myogenic potentials (RoVEMPs) can detect muscle fatigability through direct recording from EOMs. RoVEMPs, at stimulation rates of 20–30 Hz and recording from inferior oblique muscles, effectively distinguished MG patients from healthy controls (sensitivity 71–89% and specificity 64–86%) (68–70) and from patients with other neuromuscular diseases (sensitivity 67% and specificity 82%) (70). This non-invasive technique is a promising diagnostic tool and warrants confirmation in further studies.

### Response to Cholinesterase-Inhibitors

In MG patients, ChE-Is improve NMT by prolonging ACh half-life at the motor endplate. A positive response as unequivocal improvement strongly supports the diagnosis.

The infusion of the short-acting agent edrophonium chloride (max dosage 10 mg) has been used for several decades as confirmatory test. In OMG patients, clinical improvement can be readily quantified when obvious ptosis and/or severe restriction of ocular motility are present. In different studies, response rates ranged between 88 and 95% (19, 63, 71). Edrophonium injection often elicits lacrimation, sweating and fasciculations; as more serious adverse effects (AEs) as bronchoconstriction and severe bradycardia can occur, atropine should always be kept at reach. Responsiveness to ChE-Is can also be tested with neostigmine (1–2 mg, i.m.) or pyridostigmine (60 mg, orally), with clinical evaluation after 15–30 min and 45–60 min, respectively. These slow-acting ChE-Is may have a lower diagnostic sensitivity than edrophonium given the more gradual clinical effect. A positive reaction to ChE-Is is observed in most CMS and to a lesser extent in LEMS and botulism. False responses have been described in amyotrophic lateral sclerosis, peripheral neuropathy, and, rarely, in patients with mitochondrial myopathy (72) and intracranial tumors (73).

### Other Bedside Tests

The ice pack, rest, and sleep tests are particularly helpful in patients with ptosis. Clinical evaluation of ptosis and ocular motility are performed before and immediately after an ice pack has been placed over the patient's closed eyelids for 2–5 min (ice pack test), the patient has kept his/her eyelids closed for 5 min (rest test), or the patient has slept or rested for 30 min (sleep test). A positive response consists in clear-cut symptom relief. The ice pack test sensitivity was 76.9% in patients with diplopia (74) and 92–96% in those with ptosis with specificity ranging 79–98% (66, 74–76). When the effects of the ice pack test and the rest test were compared in patients with ptosis, the former produced a stronger response (77). These assessments can be safely performed in patients with contraindications to edrophonium. In a recent study, the combination of positive results of the ice pack test and SF-EMF was associated with higher specificity (66). Table 1 summarizes the sensitivity and specificity of the main diagnostic tests in OMG. Variability among studies may reflect differences in the study population.

**Table 1:** Sensitivity and specificity of diagnostic tests in ocular myasthenia gravis.

	**Sensitivity (% positivity)**	**Specificity**
Anti-acetylcholine receptor antibodies by radioimmunoassay	38^[19]^-48^[62]^-70.9^[49]^-73^[31]^	98^[63]^-100^[19]^
Repetitive nerve stimulation	16.7^[62]^-24^[19]^-33^[61]^-44^[60]^	83^[62]^-84^[19]^-94^[63]^
Single-fiber electromyography	79^[64]^-90^[19]^-94^[66]^-100^[62]^	80^[64]^-92^[63]^-100^[19]^
Repetitive ocular vestibular evoked potentials	80^[70]^-89^[68]^	64^[68]^-82^[70]^
Response to cholinesterase inhibitors	88^[19]^-92^[63]^	50^[19]^-97^[63]^
Ice-pack test	80^[19]^-86^[66]^-92^[76]^-96^[75]^	25^[19]^-79^[66]^-79^[76]^-88^[75]^

*Numbers superscripted in parenthesis are the related references*.

OMG may mimic intracranial lesions, ocular neuropathy, migraine, internuclear ophthalmoplegia, MFS without obvious ataxia and pupillary abnormalities, progressive external ophthalmoplegia, levator aponeurosis, and orbital inflammatory disease. Thyroid disease is frequently associated with OMG (78). Magnetic resonance imaging of the brain and orbits is indicated when OMG is uncertain and even in patients with established diagnosis with atypical symptoms (79).

In patients diagnosed with OMG, particularly those with AChR Abs, a chest computed tomography is mandatory to rule out a thymoma.

## Therapeutic Options

Ptosis and, even more, diplopia interfere with daily activities and impact on health-related quality of life (QoL) (80). In addition, patients are concerned about the possibility of symptom generalization and frequently ask whether this may be prevented. Clinical management is complicated by lack of Class I evidence (81).

ChE-Is are first-line treatment in nearly all OMG patients. Oral pyridostigmine (90–300 mg/day) may sufficiently relieve mild to moderate ptosis but is less effective in resolving diplopia (82, 83). ChE-Is are generally well-tolerated and AEs, mostly consisting of diarrhea, hyperhidrosis, and muscle cramps, can usually be controlled by dose adjusting. These agents do not reduce the risk for secondary generalization. In addition, patients with chronic symptomatic OMG can develop permanent ophthalmoparesis and muscle atrophy, with reduced chance of recovery (30).

Patients with unsatisfactory response to ChE-Is are candidate for immunosuppressive treatment. In retrospective studies, prednisone and prednisolone were effective in relieving symptoms, with response rates ranging 66–86% (19, 84–86) and the rate of disease progression was much lower in patients under steroids than in those receiving pyridostigmine only (41, 44, 45, 85, 86). The EPITOME trial investigated the safety and efficacy of prednisone in OMG patients with unsatisfactory response to pyridostigmine (87). The study was closed early because of slow enrollment (11 patients were randomized of the 88 planned). Although severely underpowered, this trial showed a clear superiority of prednisone over placebo, as 83% of patients receiving prednisone (and no patient on placebo) achieved the status of minimal manifestations (MM) (88). No patient progressed to GMG (87).

Oral treatment with prednisone or prednisolone is first-choice immunosuppression in patients with disabling OMG. Treatment can be started at full dosage or with an escalating regimen, but maximum doses (25–50 mg/day) are generally lower than in GMG (83, 89, 90). Once symptom control has been achieved, prednisone is slowly tapered to the lowest effective dose or withdrawal. Maintenance doses ≤5 mg/day are well-tolerated with a favorable impact on QoL (91). Prednisone is largely available and has a rapid effect, and, in OMG, the risk of “early deterioration” is not a concern. In a recent study comparing OMG response to two steroid regimes, i.e., high-dose intravenous methyl prednisolone (IVMP) and low-dose oral prednisone, IVMP was associated with faster improvement (92). This finding deserves confirmation in further studies. Steroid-sparing agents are frequently used in long-term therapy, with the same criteria and treatment regimens as in GMG (89, 90). In uncontrolled studies, azathioprine (85), mycophenolate mofetil (19, 93), and tacrolimus (94) were beneficial both in relieving symptoms and in preventing disease progression.

OMG is deemed refractory when patients do not respond to immunosuppression or require high-dose regimens with intolerable AEs (95). In these cases, treatment options are far more limited than in GMG. Plasma exchange was tried in very few patients and resulted in no benefit (19, 96). In a RCT, no response to intravenous immunoglobulin was detected in a small OMG population (97). B cell depletion with rituximab, a chimeric anti-CD20 monoclonal Ab, has gained increasing popularity in the treatment of GMG (4, 90, 98). Rituximab was very effective in the few OMG subjects treated so far (99, 100) and, although evidence is scarce, may be considered in refractory disease. Lastly, OMG patients were not included in RCTs investigating novel therapeutic options based on complement inhibition or competition with IgG for the Fc neonatal receptor (101).

Thymectomy, when feasible, is obviously indicated in thymoma patients. Conversely, the role of therapeutic thymectomy (i.e., in non-thymoma patients for the treatment of MG) has been the object of a long-standing debate. In the past decades, when the recommended surgical technique was extended trans-sternal thymectomy, it was considered a too aggressive option for a limited disease. Currently, minimally invasive surgery can make thymectomy more acceptable in this population. As available evidence comes from retrospective heterogeneous studies, no firm conclusions can be drawn on the efficacy of thymectomy in OMG. Some investigations reported no clear benefit (102–104), while others found early thymectomy to be associated with an increased probability of remission and improvement (105–108). In a metanalysis, the pooled remission rate after surgery was 50% with a better outcome in pediatric patients (108). In current practice, thymectomy is considered on an individual basis (109), as initial treatment in early-onset AChR-positive OMG (110) or for patients with unsatisfactory response to immunosuppression (83).

Supportive measures as crutch glasses for severe ptosis and prisms in patients with diplopia are helpful in treatment-resistant OMG. Ptosis surgical correction is effective and well-tolerated when exposure keratitis is avoided (90, 111); the possibility of diplopia worsening should be discussed beforehand. Topical naphazoline, a mainly α2-agonist, was found to be effective in relieving mild to moderate ptosis (112). Strabismus surgery can be considered in patients with stable ocular misalignment (113), but recurrences can complicate the long-term course.

## Conclusions

There are still considerable challenges in the diagnosis and treatment of OMG. In dSN patients, borderline results on SF-EMG and ambiguous response to ChE-Is may be misleading and the diagnostic utility of new Ab assays is not yet established. Early steroid treatment is often required to improve symptoms and may reduce disease progression. OMG prognosis is generally good in patients who can achieve and maintain symptom control with low-dose treatment, but it is much less favorable in those with relapsing disease requiring prolonged immunosuppressive therapy at high-dose regimens. At present, there are few treatment options for refractory OMG. Clinical management would greatly benefit from RCTs and prospective studies in large cohorts.

## Author Contributions

AE drafted the manuscript. RI revised the manuscript for intellectual content. Both authors contributed to the article and approved the submitted version.

## Conflict of Interest

AE is a scientific award jury member for Grifols, independent safety data monitor for UCB. The remaining author declares that the research was conducted in the absence of any commercial or financial relationships that could be construed as a potential conflict of interest.
